# Physicochemical Characterization of Electrospun Poly(vinyl alcohol)/Parsley Extract Nanofibers

**DOI:** 10.3390/polym18212715

**Published:** 2026-11-02

**Authors:** Hediye Deniz Ada

**Affiliations:** Vocational School of Kütahya Technical Sciences, Department of Chemistry and Chemical Processing Technologies, Kütahya Dumlupınar University, 43020 Kütahya, Türkiye; hdeniz.ada@dpu.edu.tr

**Keywords:** electrospinning, polyvinyl alcohol, parsley extract, nanofibers, incorporation, antioxidant activity

## Abstract

Parsley (*Petroselinum crispum*) leaves are rich in phenolic acids, flavonoids, and volatile compounds with antioxidant activity, but these bioactives degrade rapidly after extraction. Here, aqueous parsley leaf extract (PLE) was incorporated into electrospun polyvinyl alcohol (PVA) nanofibers and characterized by SEM, ATR-FTIR, XRD, thermogravimetric analysis (TGA), and the DPPH radical scavenging assay. SEM showed continuous submicron fibers under the conditions tested (17 kV, 18 cm, 0.3 mL/h), with a mean diameter of 333.7 ± 94.0 nm (*N* = 40, measured across two separate micrographs). ATR-FTIR showed that PVA and PLE bands were both present in the composite spectrum, with an O–H stretching shift consistent with possible hydrogen-bonding interactions. XRD showed that the main PVA reflection persisted after electrospinning, with a lower raw peak intensity after PLE addition, suggestive of partial interaction with the polymer chains. TGA showed that the main decomposition temperature of the PLE/PVA nanofiber (277 °C) fell between those of PVA (266 °C) and PLE (296 °C), consistent with PLE incorporation within the PVA matrix. In the DPPH assay (*n* = 3), pure PLE showed a radical scavenging activity of 96.61 ± 0.29% and the PLE/PVA nanofiber 89.32 ± 0.27%, indicating measurable antioxidant activity of the composite fibers; because the samples were not normalized to an equivalent extract dose and no PVA-only control was included, the activity retained after electrospinning is not quantified. These preliminary results show that aqueous parsley extract can be incorporated into PVA nanofibers with retention of fibrous morphology. Unlike a recently reported multicomponent PVA/metal–organic-framework system containing parsley extract for wound healing, this work addresses a simple, organic-solvent-free binary PLE/PVA system and its basic physicochemical characteristics; it does not claim a performance advantage over that or other plant extract-loaded PVA systems, and release, antimicrobial, and application-specific tests were not part of this study.

## 1. Introduction

Electrospinning is one of the most widely used techniques for the production of polymeric nanofibers capable of incorporating bioactive compounds. The resulting fibers combine a high surface area-to-volume ratio with a porous, interconnected structure, allowing for the entrapment and simultaneous gradual release of sensitive molecules within the fiber matrix. Owing to these properties, nanofibers produced by electrospinning are widely used in drug delivery, wound care, tissue engineering, and more recently, in active food packaging [1,2].

Among the polymers used in electrospinning, polyvinyl alcohol (PVA) is one of the most frequently chosen due to its water solubility, biocompatibility, non-toxicity, and favorable mechanical behavior [3]. The abundance of hydroxyl groups allows PVA to physically interact with a wide variety of bioactive substances through hydrogen bonding. PVA-based nanofibers loaded with medicinal or culinary plant extracts have been repeatedly reported to maintain or even enhance the functional properties of the loaded material. For example, extract-loaded PVA dressings have been shown to improve wound healing performance [4,5]. Because of this versatility, PVA is a suitable and low-cost matrix for incorporating natural antioxidants that are difficult to stabilize in their free forms.

Parsley (*Petroselinum crispum*) is an Apiaceae plant consumed worldwide as both food and traditional medicine. Its leaves are a rich source of phenolic acids, flavonoids (including apigenin and its glycoside apiin), and volatile components such as apiol and myristicin. Together, these compounds confer significant antioxidant and antimicrobial activity to parsley [6,7]. Despite this promising phytochemical profile, bioactive substances derived from parsley degrade rapidly after extraction due to their sensitivity to light, oxygen, and temperature. Therefore, incorporating them into a protective polymeric matrix offers a practical way to preserve their functional properties over time [2].

Many studies have described the electrospinning process of PVA together with essential oils, seed mucilages, or other plant-derived extracts [8,9,10,11,12,13,14,15,16,17,18,19]. Very recently, one study reported the incorporation of parsley (*Petroselinum crispum*) extract into electrospun PVA nanofibers in combination with Zn-ascorbate metal–organic frameworks (MOFs), targeting antibacterial wound-healing applications and reporting antioxidant and antibacterial performance for the resulting three-component MOF/PLE/PVA system [20]. This recent report indicates that combining parsley extract with electrospun PVA nanofibers is not, by itself, an unprecedented strategy; however, that study focuses on a complex, multicomponent formulation designed for a specific biomedical application, in which the individual contribution of the aqueous parsley extract to the physicochemical behavior of the fiber matrix is not isolated from that of the co-incorporated MOF. In contrast, to our knowledge, a simple, single-component, organic-solvent-free aqueous PLE/PVA binary system has not been systematically characterized in terms of its basic physicochemical, structural, thermal, and antioxidant behavior. Therefore, this study aims to produce PVA nanofibers loaded with aqueous parsley leaf extract by electrospinning and to characterize the resulting fibers in terms of morphology, chemical structure, crystallinity, thermal stability, and antioxidant activity, providing basic physicochemical data for this simpler binary system. No performance advantage over the MOF-containing system or over other plant extract-loaded PVA nanofibers is claimed or demonstrated here, and the study does not target a specific application (such as wound dressing, food packaging, or antioxidant delivery): it is a preliminary physicochemical characterization and a first antioxidant (DPPH) screening of this particular formulation. Scanning electron microscopy (SEM) was used to examine the fiber morphology. ATR-FTIR spectroscopy was used to evaluate the chemical integrity of the composite system and possible interactions between PVA and PLE. X-ray diffraction (XRD) was used to evaluate the crystal structure of the obtained nanofibers.

Similar strategies have been employed with other plant- and fruit-derived antioxidant extracts in PVA and related hydrophilic polymers. PVA nanofibers loaded with jujube extract extended the post-harvest shelf life of strawberries thanks to a combination of antioxidant and antimicrobial effects [21]. PVA/PVP composite nanofibers loaded with *Nervilia fordii* extract achieved encapsulation efficiencies of over 90% and delayed lipid oxidation in packaged foods [22]. Poly(ethylene oxide) fibers loaded with aloe vera agricultural waste extract retained their antioxidant activity after electrospinning, but experienced some loss in thermal stability [23]. Polyvinylpyrrolidone/cellulose acetate fibers loaded with garlic extract and glycerin improved fiber utilization while maintaining antibacterial activity [24]. Parsley extract alone has been shown to have significant antioxidant and antibacterial activity in its unencapsulated form [25]. PVA nanofibers loaded with other polyphenol-rich extracts such as black tea have shown sustained antioxidant release under different food simulation conditions [26]. These reports suggest that PVA is a plausible matrix for plant extracts such as the parsley extract studied in this work; the suitability of PVA for parsley extract specifically was not evaluated beyond the characterizations reported here.

## 2. Materials and Methods

### 2.1. Materials

Fresh parsley (*Petroselinum crispum*) leaves were purchased from local markets in Kütahya, Turkey. Polyvinyl alcohol (PVA, mean molecular weight 88,000 g/mol; a mid-spacing molecular weight was selected as a practical starting point offering a favorable balance between solution viscosity and spinnability for aqueous electrospinning, consistent with previous reports using PVA in this molecular weight range [3,4]) was supplied by Acros Organics (Geel, Belgium) and used as a synthetic polymer matrix. According to the supplier’s specification, the PVA grade used was 98.0–98.8% hydrolyzed; this value was not independently verified by the authors, and the degree of hydrolysis can influence both crystallinity and FTIR carbonyl-region features (Section 3.2 and Section 3.3). All aqueous solutions, including the PLE extraction medium and the PVA solvent, were prepared using ultrapure water (resistivity 18.2 MΩ·cm) obtained from the Kütahya Dumlupınar University Advanced Technologies Application and Research Center (DPU-İLTEM).

### 2.2. Preparation of Parsley Leaf Extract (PLE)

Parsley leaves were washed with ultrapure water, air-dried at room temperature, and finely chopped. Twenty grams of dried leaves were combined with 200 mL of ultrapure water and stirred continuously for 20 min at 90 °C. The mixture was cooled to room temperature and filtered through filter paper (coarse, qualitative grade) to remove gross plant debris, yielding a visually clear aqueous parsley leaf extract (PLE). This filtration step removes only coarse cellular fragments and does not constitute purification of the extract; PLE therefore remains a crude, multi-component aqueous mixture rather than an isolated active compound and was used without further purification or fractionation [27]. This approach was adopted deliberately, in line with the study’s aim of evaluating a simple, low-cost, whole-extract system rather than an isolated bioactive fraction; however, we acknowledge that the purity, exact composition, and possible presence of residual fine cellular material in PLE were not independently verified (e.g., by centrifugation, membrane filtration, or compositional analysis), and this is noted as a limitation of the present study.

### 2.3. Preparation of Polymer Raw Material

A 10% (*w*/*w*) PVA solution was prepared by dissolving the polymer in ultrapure water at 80 °C for 2 h under continuous magnetic stirring until a homogeneous and bubble-free solution was obtained. The electrospinning raw material was prepared by mixing PLE with the PVA solution in a volume ratio of 20:80 (PLE:PVA); this ratio was chosen as the composition that gave the most consistent and well-formed fibers as a result of preliminary trials. Based on this mixing ratio and the 10% (*w*/*w*) PVA solution concentration, and assuming a solution density close to 1 g/mL, the final electrospinning feedstock contained approximately 8% (*w*/*w*) PVA on a total-mixture basis. The dry-solids content of the extract was determined gravimetrically on a separately prepared batch of PLE (similar preparation procedure; the batch used for spinning was not retained): three 5 mL aliquots were dried in pre-weighed dishes at 60 °C for approximately 5 h until completely dry and then weighed, giving 75.3 ± 2.0 mg of solids per 5 mL, i.e., 15.1 ± 0.4 mg/mL (*n* = 3). On this basis, the 20 mL of PLE in each 100 mL of feedstock contributes approximately 0.30 g of extract solids, compared with approximately 8 g of PVA, corresponding to a dry PLE:PVA mass ratio of roughly 4:100 (about 3.6% of the total dry solids); the volumetric ratio should therefore not be read as the dry mass ratio. This is an estimate based on a re-prepared extract, so batch-to-batch variation is possible, and the actual loading efficiency in the fiber mat was not measured; its quantification (e.g., by extraction and spectrophotometric determination of a marker compound) is identified as a direction for future work.

### 2.4. Nanofiber Production by Electrospinning Method

Electrospinning was performed using a commercial electrospinning device (NL-MS/K, Nanolysis, Ankara, Turkey), which consists of a high-voltage power supply, a syringe pump, a capillary needle-tip syringe, and a collector unit. A grounded, stationary flat aluminum foil sheet served as the collector. A 5 mL disposable plastic syringe (pharmacy-grade), fitted with a stainless-steel needle (21–23 gauge), was used to deliver the polymer solution—consistent with the syringe volume and stationary, grounded aluminum-foil collector previously reported for the same electrospinning device used within this laboratory to fabricate other PVA-based composite nanofibers [28]. The needle had an internal diameter of approximately 0.5 mm, and the needle tip was connected to the positive terminal of the high-voltage supply. Fibers were collected over a total deposition time of approximately 1 h for each formulation. Solution viscosity and electrical conductivity were not measured, and the ambient temperature (approximately 20–25 °C) and relative humidity (approximately 30–50%) reflect the laboratory conditions, which were not regulated or continuously logged; these parameters will be documented in future work to support reproducibility. The process parameters selected for the PLE/PVA (20:80, *v*/*v*) feedstock, an applied voltage of 17 kV, a tip-to-collector distance of 18 cm, and a solution flow rate of 0.3 mL/h, were chosen from a limited number of preliminary trials judged qualitatively rather than by a systematic optimization procedure. The conditions explored are summarized in Table 1: the ranges denote the span examined within each PLE:PVA ratio. Trials 1–4 were evaluated qualitatively and their trial-by-trial outcomes were not recorded, so no comparison between them is made, and trial 5 lists the final conditions used for all characterizations. Only the 20:80 (*v*/*v*) formulation was characterized in detail; the influence of PLE concentration on fiber morphology, stability, and antioxidant activity will be addressed in future work.

### 2.5. Characterization of Nanofibers

#### 2.5.1. Surface Morphology (SEM)

The morphology and diameter of nanofibers produced by the electrospinning method were examined by scanning electron microscopy (Nova NanoSEM 650, FEI, Hillsboro, OR, USA) using a concentric backscattering (CBS) detector at an acceleration voltage of 10 kV. Before imaging, the samples were coated with a thin gold–palladium layer to make them conductive. Fiber diameter was measured using the straight-line tool in ImageJ 1.54g (NIH, Bethesda, MD, USA), calibrated against the scale bar of each micrograph. A total of 40 measurements were obtained from two separate micrographs (10,000× and 20,000× magnification), collected across separate measurement sessions. Each measurement was taken from a visually distinct, non-overlapping fiber segment, away from fiber junctions, yielding 40 distinct fiber-diameter observations from a single electrospun mat (distinct fibers, but not independently prepared samples). This sample size remains below the ideal target of 50–100 independent measurements, and measurement of a larger number of distinct fibers across additional, independently prepared samples is recommended for future work.

#### 2.5.2. Chemical Analysis (ATR-FTIR)

To evaluate the chemical interactions between the PLE and PVA matrix, Fourier transform infrared spectroscopy was performed with an Alpha spectrometer (Bruker Optik GmbH, Ettlingen, Germany) in attenuated total reflection (ATR) mode using a diamond ATR crystal, with a resolution of 4 cm^−1^ in the range of 500–4000 cm^−1^.

#### 2.5.3. Crystallographic Structure (XRD)

The crystal properties of the nanofibers were analyzed by X-ray diffraction (Empyrean diffractometer, Malvern Panalytical, Almelo, The Netherlands) using Cu Kα radiation (λ = 0.154 nm) at 40 kV. The 2θ range was scanned between 5° and 70° with a scan rate of 1°/min. For pure PVA, the material analyzed by XRD was the electrospun PVA nanofiber mat (i.e., the same physical form as the PLE/PVA nanofiber sample), rather than the starting PVA powder or a solvent-cast film; sample mass, thickness, and packing were not independently controlled or normalized between the PVA and PLE/PVA measurements.

#### 2.5.4. Thermal Analysis (TGA)

Thermogravimetric analysis was performed using a simultaneous thermal analyzer (NEXTA STA200, Hitachi High-Tech, Tokyo, Japan), capable of concurrent TG and DTA measurement, at the Advanced Technology Application and Research Center (ARUM), Eskişehir Osmangazi University, Türkiye, under a nitrogen (N_2_) atmosphere (flow rate 200 mL/min) in platinum crucibles, with a heating rate of 10 °C/min from room temperature to approximately 715 °C. The initial sample masses, as reported by the analysis facility, were 4.019 mg for pure PVA, 5.983 mg for pure PLE, and 8.020 mg for the PLE/PVA nanofiber; the masses were therefore not matched between samples, and no blank-crucible baseline or buoyancy correction was applied to any of the three curves. Derivative thermogravimetry (DTG) curves were derived from the TG curves, and differential thermal analysis (DTA) curves were recorded simultaneously with the TG curves. The apparent mass increase (up to approximately 104%) observed for pure PVA below 150 °C, discussed in Section 3.4, was not corrected using an independent baseline run; consequently, the absolute weight-loss values reported for pure PVA in this temperature range should be interpreted with appropriate caution, and a baseline-corrected repeat measurement is recommended for future quantitative work.

#### 2.5.5. DPPH Radical Scavenging Assay

The antioxidant activity of the pure parsley leaf extract (PLE) and the electrospun PLE/PVA nanofiber was evaluated using the 2,2-diphenyl-1-picrylhydrazyl (DPPH) radical scavenging assay [25]. A 0.1 mM methanolic DPPH solution was prepared, and samples were mixed with this solution. Mixtures were incubated in the dark at room temperature for 30 min, after which the absorbance was measured at 517 nm using a UV-Vis spectrophotometer (UV-1700 PharmaSpec, Shimadzu, Kyoto, Japan). Each measurement was performed in triplicate (*n* = 3), and results are reported as mean ± standard deviation (SD). The percentage of DPPH radical scavenging activity was calculated as: % inhibition = [(A(control) − A(sample))/A(control)] × 100, where A(control) is the absorbance of the control solution (without sample) and A(sample) is the absorbance of the tested sample at 517 nm. No extract-free (PVA-only) nanofiber control, reference antioxidant (e.g., ascorbic acid), or normalization of the samples to an equivalent dry extract dose was included in this assay, and the release of active compounds from the fibers into the DPPH medium was not examined.

## 3. Results and Discussion

Nanofibers were successfully produced by combining parsley leaf extract (PLE) with PVA using the electrospinning method. Approximately 50 g of fresh parsley leaves yielded approximately 20 g of dry plant material after drying. The extract obtained from this dry material was then incorporated into PVA-based nanofibers using the electrospinning method. All characterizations reported below were performed on a single PLE preparation and a single electrospun mat; the DPPH measurements are technical triplicates of this sample, and batch-to-batch reproducibility was not assessed.

### 3.1. Nanofiber Morphology

The diameter and surface morphology of nanofibers produced by electrospinning are important for their intended applications. Both are affected by the electrospinning process parameters and the physico-chemical properties of the spinning solution.

**Figure 1:**
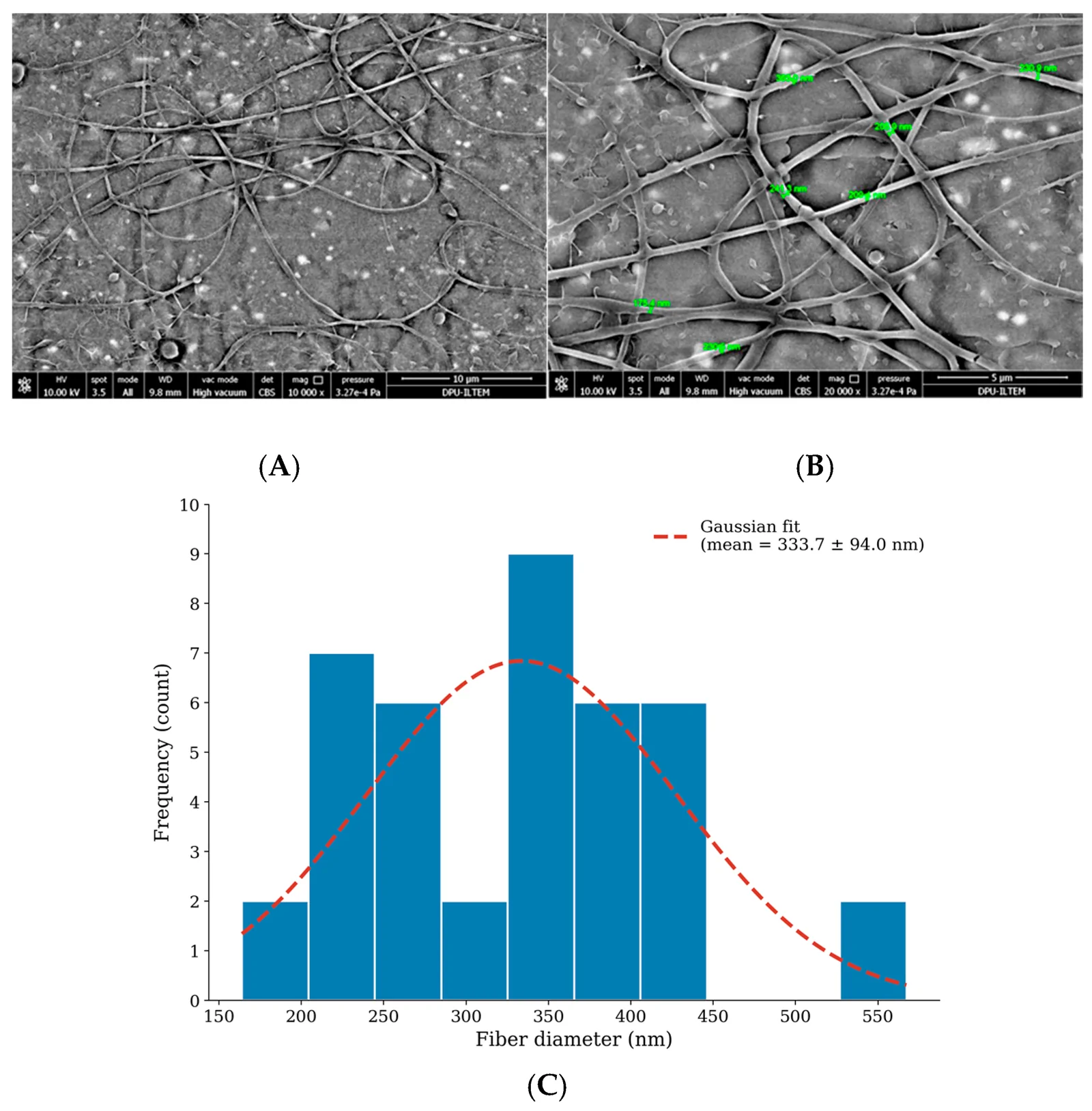
Scanning electron microscope (SEM) images of PLE/PVA nanofibers produced by electrospinning method, recorded at (**A**) 10,000× and (**B**) 20,000× magnification, and (**C**) histogram of the fiber diameter distribution (*N* = 40), combining measurements from both micrographs and all measurement sets, with a fitted Gaussian curve. The green labels in (**B**) indicate the fiber diameters (nm) measured directly on the micrograph.

SEM micrographs of PLE/PVA nanofibers produced by electrospinning were taken at 10,000× and 20,000× magnification (Figure 1). Under the processing conditions used in this study (17 kV, 18 cm, 0.3 mL/h), continuous submicron fibers were obtained, together with a background film-like region and occasional localized bead-like thickenings, particularly visible at higher magnification. This background feature is most likely attributable to incomplete solvent evaporation and partial fiber fusion during deposition rather than to electrospraying, since discrete, continuous fiber segments with a defined diameter are still clearly resolved throughout the micrographs; a minor electrosprayed fraction cannot be fully excluded, and this could be checked in future work using longer collector distances or lower solution concentrations. Other plant-extract-loaded PVA electrospun nanofiber systems, including several discussed above [8,9,16,18], have generally been reported to give smooth, continuous control fibers without an extensive film-like background; this is consistent with, but does not on its own confirm, the interpretation that the background feature observed here relates mainly to the incorporated PLE rather than to the PVA matrix itself. The fibers are submicron in width, with a mean diameter of 333.7 ± 94.0 nm (*N* = 40), based on measurements combined from two separate micrographs and separate measurement sessions (Figure 1A,B; see Section 2.5.1), with the corresponding diameter distribution shown in Figure 1C. Each measurement was taken from a visually distinct, non-overlapping fiber segment, yielding 40 distinct fiber-diameter observations from a single electrospun mat (distinct fibers, but not independently prepared samples). Mean diameter estimates differed somewhat between the measurement sets (237 nm for the 7-fiber set, 349 nm for the 20-fiber set and 363 nm for the 13-fiber set measured subsequently), reflecting the sensitivity of manual measurement to local sampling and operator judgment; the pooled estimate is reported here as the most representative value. A larger dataset (ideally 50–100 fibers across multiple micrographs and independently prepared samples) would give a more robust diameter distribution and is planned for future work. In preliminary trials, a higher applied voltage and flow rate (20 kV, 0.5 mL/h) resulted in more pronounced bead formation along the fiber axis; this is most likely due to a local mismatch between the applied voltage and the viscosity/conductivity of the PLE-containing spinning solution, an effect frequently observed when these parameters are not well matched to the raw material composition. Reducing the applied voltage and flow rate to 17 kV and 0.3 mL/h, respectively, without changing the tip-to-collector distance, visibly reduced bead formation relative to these preliminary trials, though it was not entirely eliminated. These voltage- and flow-rate-dependent trends are consistent with reports for other plant-extract-loaded PVA systems [8,9,16,18]; the conditions used were selected from a limited number of qualitative trials. Beaded fibers have likewise been reported for PVA fibers loaded with other plant extracts, particularly at higher extract contents [29], and beads are generally attributed to insufficient chain entanglement of the spinning solution [30]. Because only two micrographs were analyzed and no PVA-only micrograph was obtained, the uniformity of the morphology across the entire mat and the origin of the film-like background (PLE, PVA, or the processing conditions) cannot be fully resolved from the present data.

### 3.2. FTIR Spectra

**Figure 2:**
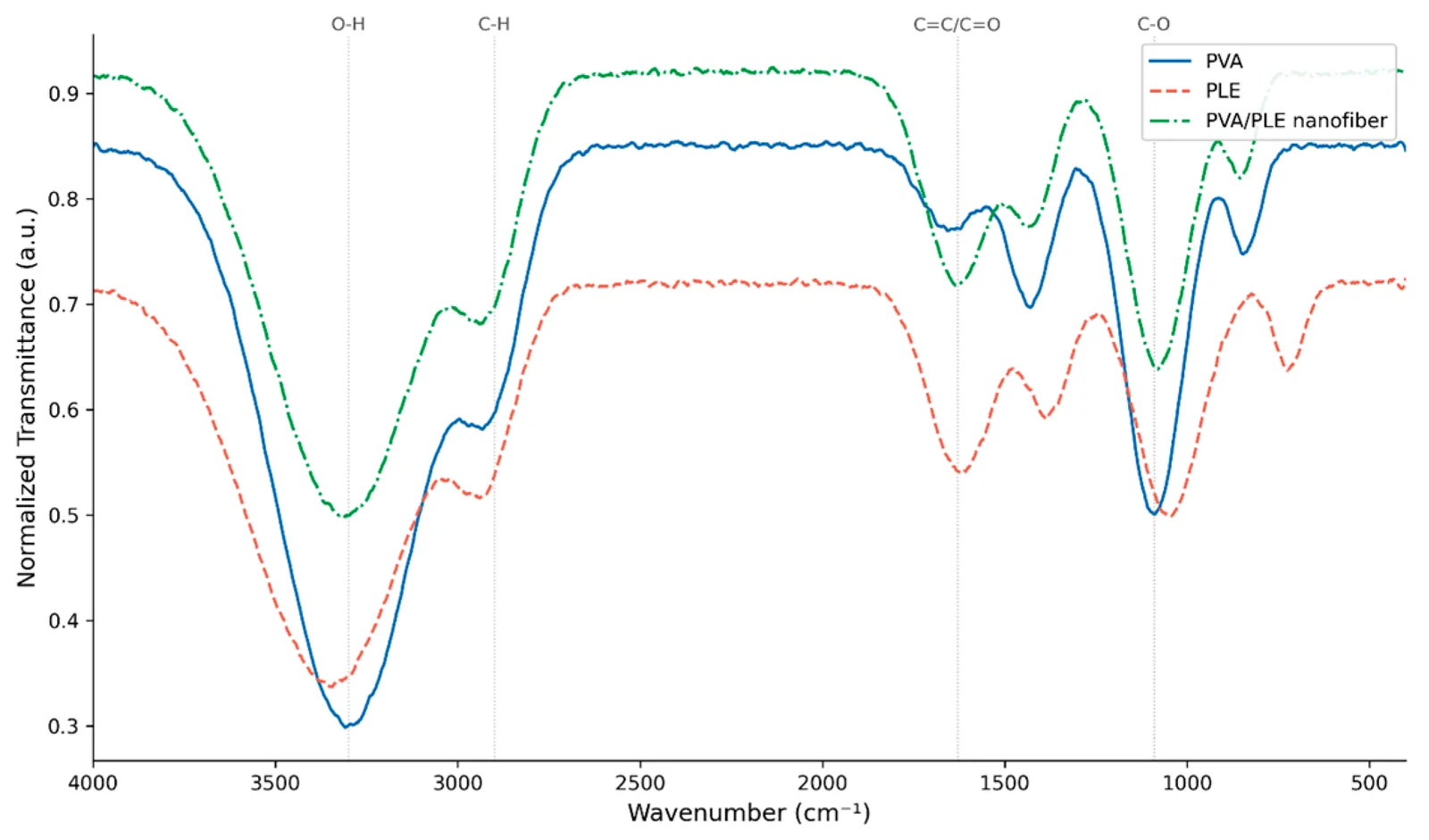
FTIR spectra of pure PVA, parsley leaf extract (PLE), and electrospinning-produced PLE/PVA nanofiber.

FTIR spectroscopy provides information about possible interactions between functional groups in a material and the components of a composite system. In this study, FTIR spectra of pure PVA, pure PLE, and electrospinning-produced PLE/PVA nanofibers were recorded in the range of 500–4000 cm^−1^ (Figure 2). Band assignments for all three samples are summarized in Table 2.

The spectrum of pure PVA showed major absorption bands at 3323.2, 2122.5, 1636.8 and 567.8 cm^−1^. The broad band at 3323.2 cm^−1^ is attributed to O–H stretching originating from the hydroxyl groups of PVA. The weak band at 2122.5 cm^−1^ does not correspond to the conventional aliphatic C–H stretching region (typically 2800–3000 cm^−1^); given its low intensity and position, it is more likely associated with a minor instrumental or atmospheric (CO_2_) interference band rather than a diagnostic vibration of PVA and is therefore not further interpreted here. The band at 1636.8 cm^−1^ is tentatively attributed to the H–O–H bending vibration of physically absorbed water, which is commonly observed in hygroscopic PVA films and powders; a contribution from residual carbonyl groups cannot be ruled out, since the supplier-stated degree of hydrolysis (98.0–98.8%) was not independently verified, although the residual acetate content is expected to be low and carbonyl stretching is typically expected at higher wavenumbers (1700–1740 cm^−1^). The band at 567.8 cm^−1^ is attributed to C–C skeletal vibrations, consistent with previously reported PVA spectra [8,9].

The spectrum of pure PLE showed absorption bands at 3321.8, 2126.7, 1636.8, 1097.3 and 604.6 cm^−1^. The broad band around 3321.8 cm^−1^ reflects hydroxyl groups from phenolic compounds and polysaccharides naturally present in parsley leaves. PLE was analyzed in liquid (aqueous extract) form, deposited and air-dried on the ATR crystal prior to measurement. Overall, the PLE spectrum shares several broad features with the PVA spectrum described above, which is not unexpected given that both materials are rich in hydroxyl-bearing, hydrogen-bonding functional groups; the 1636.8 cm^−1^ band in particular is again most consistent with absorbed water (H–O–H bending) rather than a compound-specific fingerprint, and the overall similarity to PVA should be interpreted with appropriate caution rather than as strong evidence for a distinct PLE identity. The band at 1097.3 cm^−1^ is tentatively attributed to C–O stretching, potentially associated with ether and ester bonds found in flavonoids and other polyphenolic components of parsley, although this region also overlaps with PVA’s own C–O stretching vibrations and is therefore not fully diagnostic on its own [8,9,10,11].

For PLE/PVA nanofibers, characteristic bands were detected at 3314.7, 2123.9, 1636.8, 1100.2 and 589.0 cm^−1^. Two observations are noted. First, the O–H stretching band shifted from 3323.2 cm^−1^ in pure PVA to 3314.7 cm^−1^ in the composite fiber; this shift alone is not sufficient to firmly establish new hydrogen-bonding interactions between PVA and PLE and is better described as consistent with possible hydrogen-bonding interactions between the hydroxyl groups of PVA and the phenolic components of PLE, rather than definitive proof of such interactions. Secondly, a band at 1100.2 cm^−1^, in a spectral region associated with both PLE and PVA C–O stretching vibrations, is present in the nanofiber spectrum; while consistent with the incorporation of PLE into the fiber, this band cannot be unambiguously assigned to PLE alone given the spectral overlap with PVA discussed above. Overall, these FTIR results are consistent with the incorporation of PLE within the PVA fiber matrix; because neither the spatial distribution of PLE within the fibers nor a specific new chemical bond was directly measured, the more conservative terms “incorporation” or “loading” are therefore used throughout this manuscript. Broadly similar spectral overlap between plant-extract and PVA matrix components has been reported for other plant extract-loaded PVA nanofiber systems [9,12,13,15,31].

### 3.3. XRD Analysis

**Figure 3:**
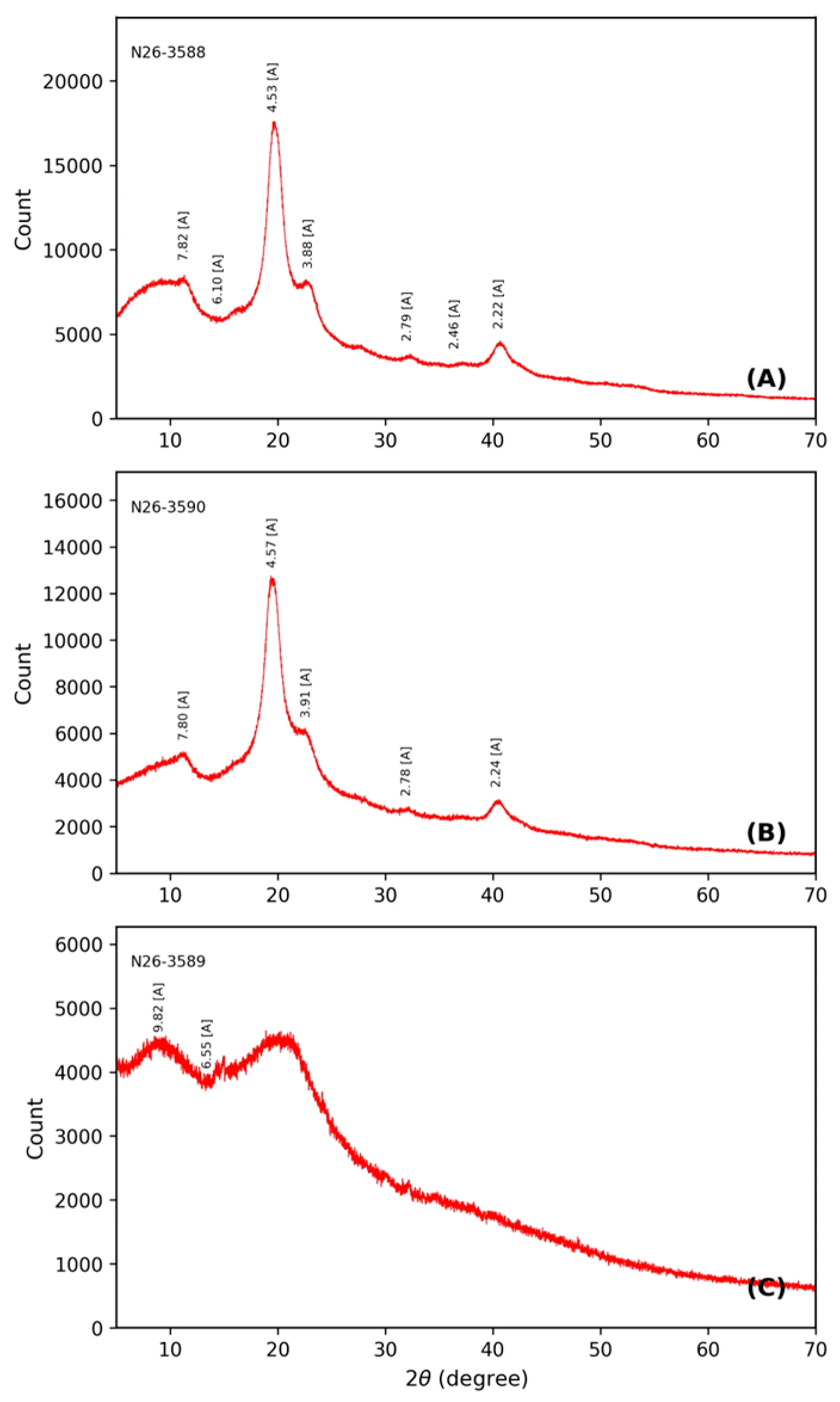
X-ray diffraction (XRD) patterns of (**A**) pure PVA, (**B**) electrospinning-produced PLE/PVA nanofiber, and (**C**) pure PLE. Numbers above the peaks give the interplanar spacing d in Å ([A] = Å).

X-ray diffraction was used to investigate the crystal structure of pure PVA, pure PLE, and PLE/PVA nanofibers produced by electrospinning over the 2θ range of 5–70° (Figure 3). Peak positions and crystallographic assignments for all three samples are summarized in Table 3.

The diffractogram of pure PVA (Figure 3A) showed a sharp, intense peak at 2θ ≈ 19.6° (d-spacing ≈ 4.53 Å), attributed to the crystallographic plane of the PVA monoclinic lattice (101). This reflection originates from strong internal and external molecular hydrogen bonds between hydroxyl groups in adjacent PVA chains, allowing the polymer to form a partially ordered, quasi-crystalline structure. In addition, several weak reflections were observed at 2θ ≈ 11.3°, 14.5°, 22.9°, 32.1°, 36.5° and 40.6°, consistent with patterns previously reported for semi-crystalline PVA.

In contrast, pure PLE (Figure 3C) did not show sharp crystalline reflections. Instead, two broad bumps centered around 2θ ≈ 9° and 2θ ≈ 20° were observed; this is characteristic of an essentially amorphous material—an expected result, since PLE is a complex mixture of phenolic compounds, flavonoids and other plant-derived components that lack long-range structural ordering. The diffractogram of the PLE/PVA nanofiber (Figure 3B) showed that the main PVA reflection was still present after electrospinning, at 2θ ≈ 19.4° (d ≈ 4.57 Å). The raw peak intensity of this reflection was lower (approximately 17,600 counts for pure PVA versus 12,700 counts for the PLE/PVA nanofiber) and the peak was slightly broadened compared to pure PVA. Because sample mass, thickness, and packing were not normalized between measurements, this difference in raw counts is not a quantitative measure of crystallinity; qualitatively, however, the persistence of the main reflection together with its reduced intensity and broadening is suggestive of a partial disruption of the ordered packing of the PVA chains, tentatively attributed to hydrogen-bonding interactions between the hydroxyl groups of PVA and the phenolic components of PLE (consistent with the FTIR results in Section 3.2). Quantifying crystallinity from normalized peak areas is a natural next step. Similar qualitative reductions in apparent PVA crystallinity after the addition of plant extracts or other biopolymers have been reported previously and are generally attributed to disruption of intermolecular hydrogen bonds within the PVA matrix and to the rapid solvent evaporation during electrospinning, which limits the time available for chain ordering [4]. Together with the FTIR results, these XRD observations are consistent with physical incorporation of PLE within the PVA matrix without the formation of new crystalline phases.

When the SEM, FTIR and XRD results are considered together, they give a broadly consistent picture of the PLE/PVA nanofiber system. SEM shows that continuous submicron fibers are obtained together with some localized bead-like and film-like features, indicating that PLE does not prevent fiber formation from the PVA solution under the conditions tested. The shift in the O–H stretching band in the FTIR spectrum is consistent with hydrogen-bonding interactions between PVA and PLE, and the XRD results are compatible with this interpretation at the structural level: the persistence of the PVA (101) reflection together with its reduced raw intensity suggests that PLE is incorporated into the PVA matrix without disrupting fiber formation, while potentially interacting with the polymer chains in a way that limits their crystalline packing. Taken together, these findings are consistent with incorporation of PLE within the PVA nanofiber structure without evidence of major new crystalline phases; a quantitative confirmation of the crystallinity comparison and of the molecular-level compatibility of the two components are the next steps.

### 3.4. Thermal Stability (TGA)

Thermogravimetric analysis (TGA) was used to evaluate the thermal stability and degradation behavior of pure PVA, pure PLE, and PLE/PVA nanofiber produced by electrospinning. As noted in Section 2.5.4, measurements were carried out at the Advanced Technology Application and Research Center (ARUM), Eskişehir Osmangazi University, Türkiye, under a nitrogen (N_2_), i.e., inert, atmosphere; this means the observed weight-loss steps reflect pyrolytic (non-oxidative) degradation rather than combustion, which is also why a substantial carbonaceous residue remains at the end of the scan instead of the near-complete mass loss expected under an oxidizing (air) atmosphere. Initial and maximum degradation rate temperatures for water evaporation, main thermal degradation, and carbonic residue decomposition, along with corresponding weight loss values, are reported in Table 4.

**Figure 4:**
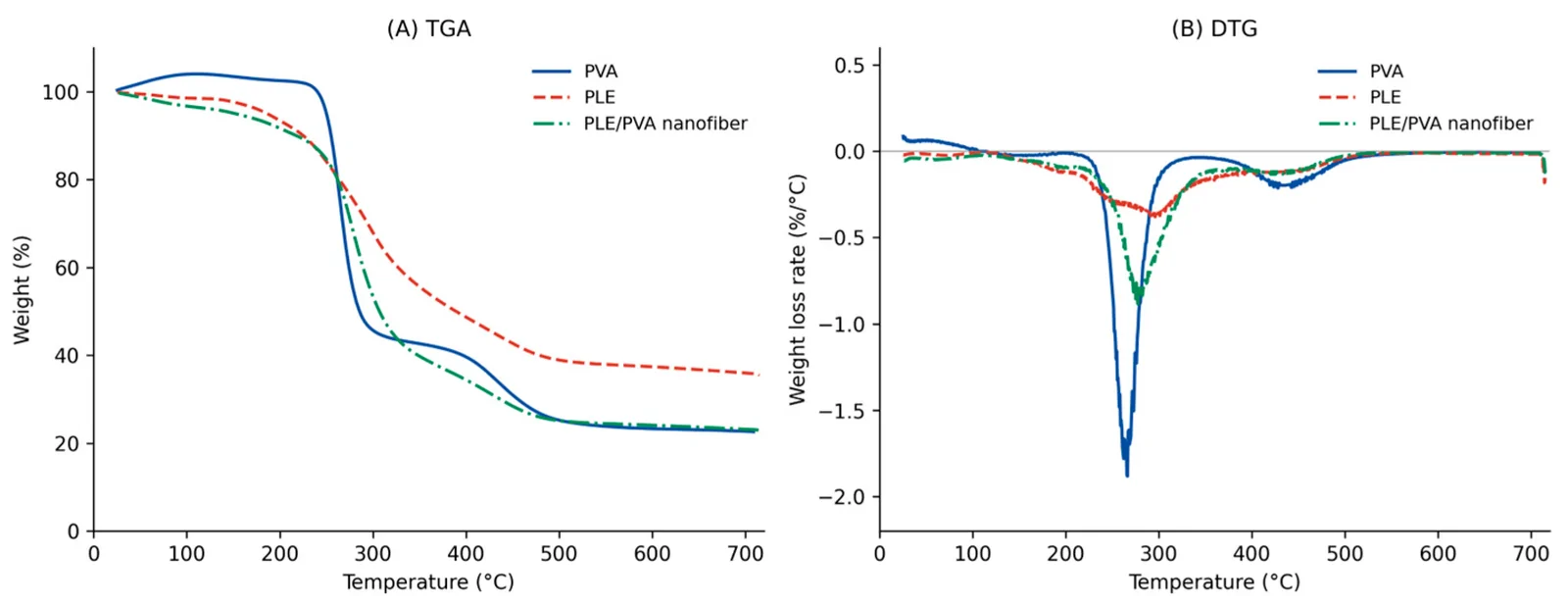
(**A**) TGA and (**B**) DTG curves of pure PVA, pure PLE and PLE/PVA nanofiber produced by electrospinning method.

**Figure 5:**
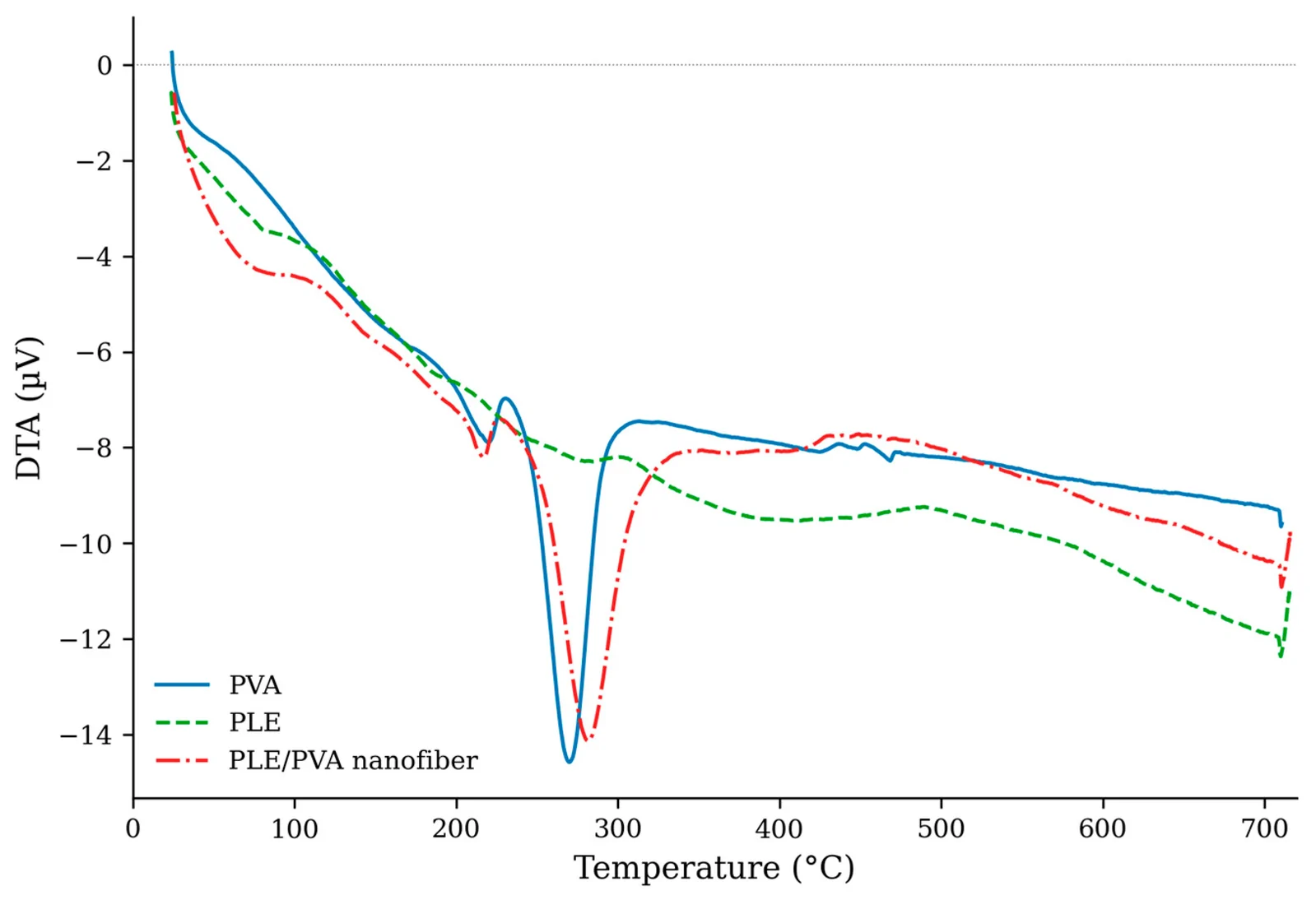
DTA curves of pure PVA, pure PLE, and PLE/PVA nanofiber produced by electrospinning method, recorded simultaneously with the TG curves.

The TGA and DTG curves of pure PVA, pure PLE, and electrospinning-produced PLE/PVA nanofiber are shown in Figure 4. Pure PVA showed an apparent increase in recorded mass (up to approximately 104%) instead of a moisture-loss step below 150 °C. This uncorrected rise (about 165 µg for the 4.019 mg sample) was tentatively attributed to a buoyancy effect caused by the decreasing density of the purge gas on heating, which has been reported to produce apparent mass gains of the order of 50–200 µg in thermogravimetric measurements and is normally removed by subtracting a blank curve [32]; however, since no independent baseline run was performed to verify this explanation (Section 2.5.4), this interpretation remains provisional, and the absolute weight-loss values reported for pure PVA below 150 °C should be treated with corresponding caution (Table 4). In contrast, both PLE and PLE/PVA nanofibers lost mass gradually and continuously from the beginning of the scan; this is consistent with the loss of bound water and low molecular weight volatile substances naturally present in the plant extract. The main degradation step occurred as a single, dominant weight loss step for all three samples. These three samples differed distinctly in the rate, sharpness, and onset temperature of this step. Pure PVA showed the most intense and narrowest degradation peak; the maximum weight loss rate occurred at 266 °C, and 63.8% of the weight loss was attributed to chain breakage and deacetylation of the PVA backbone. Pure PLE degraded more gradually over a wider temperature range; it showed a lower peak rate and smaller weight loss (49.0%) at a higher temperature (296 °C), consistent with the more complex, multi-component structure of the plant extract. This interpretation is further supported by the simultaneously recorded DTA curves (Figure 5): pure PLE showed only a weak, broad endothermic feature without a sharp, well-defined thermal event in this temperature region, consistent with its gradual, multi-stage degradation behavior. The PLE/PVA nanofiber exhibited intermediate behavior, with the main degradation peak (277 °C) and onset temperature falling between the values of the two pure components. Notably, the main degradation temperature of the composite (277 °C) was higher, not lower, than that of pure PVA (266 °C); however, XRD showed a reduction in raw PVA (101) peak intensity upon addition of PLE (Section 3.3). The DTA curves of both pure PVA and the PLE/PVA nanofiber showed a distinct, localized endothermic minimum closely coinciding with their respective main degradation peaks (at approximately 270 °C and 282 °C, respectively, consistent with the 266 °C and 277 °C DTG maxima), supporting the assignment of the main weight-loss step to an endothermic, energy-absorbing chain-scission and deacetylation process in both materials. These two observations are not necessarily contradictory, but the mechanistic link between them is speculative rather than established: one plausible explanation is that PLE-related hydrogen bonding partially disrupts long-range crystalline packing (detected by XRD) while locally requiring comparable or greater energy to break than the PVA–PVA hydrogen bonds it replaces (reflected in the shifted TGA onset), but this explanation has not been independently verified in the present study and should be treated as a hypothesis for future confirmation rather than a demonstrated mechanism. This shift and broadening of the main degradation step, together with the substantial associated weight loss (60.7%), is consistent with incorporation of PLE within the PVA matrix, but does not, on its own, establish that PLE is molecularly dispersed or that a true single-phase system has formed; this observation should be interpreted alongside the FTIR and XRD results (Section 3.2 and Section 3.3), which are similarly indicative rather than conclusive on this point.

Above 400 °C, pure PVA and PLE/PVA nanofiber showed a resolvable secondary degradation peak at 428 °C and 420 °C, respectively, attributed to further carbonization of residual polyene structures formed during the main degradation stage. Pure PLE did not show a comparable secondary peak and instead continued to lose mass slowly and steadily until the end of the scan without reaching a stable plateau. At the end of the scan (~710–716 °C), the final residual masses were 22.6% for PVA, 35.3% for PLE, and 22.8% for PLE/PVA nanofiber. The residual mass of the composite nanofiber closely matched that of pure PVA, rather than being an intermediate value between PVA and PLE; this indicates that PVA (80% by volume), the majority component of the raw material, dominates the high-temperature degradation behavior of the composite. The stepwise weight-loss values listed in Table 4 for pure PVA (0.0% + 63.8% + 17.1%) sum to approximately 81%, which does not fully reconcile with the measured final residual mass of 22.6%; this gap is consistent with the uncorrected sub-150 °C buoyancy-related artifact discussed above (Section 2.5.4), so the pure-PVA stepwise values are best read semi-quantitatively. Overall, the TGA results are consistent with the SEM, FTIR, and XRD findings in supporting the incorporation of PLE within the PVA nanofiber matrix and indicate a compatible PLE/PVA system; compositional or spatial mapping, and a baseline-corrected repeat measurement, would allow a more detailed description of the phase structure.

### 3.5. Antioxidant Activity (DPPH Assay)

**Figure 6:**
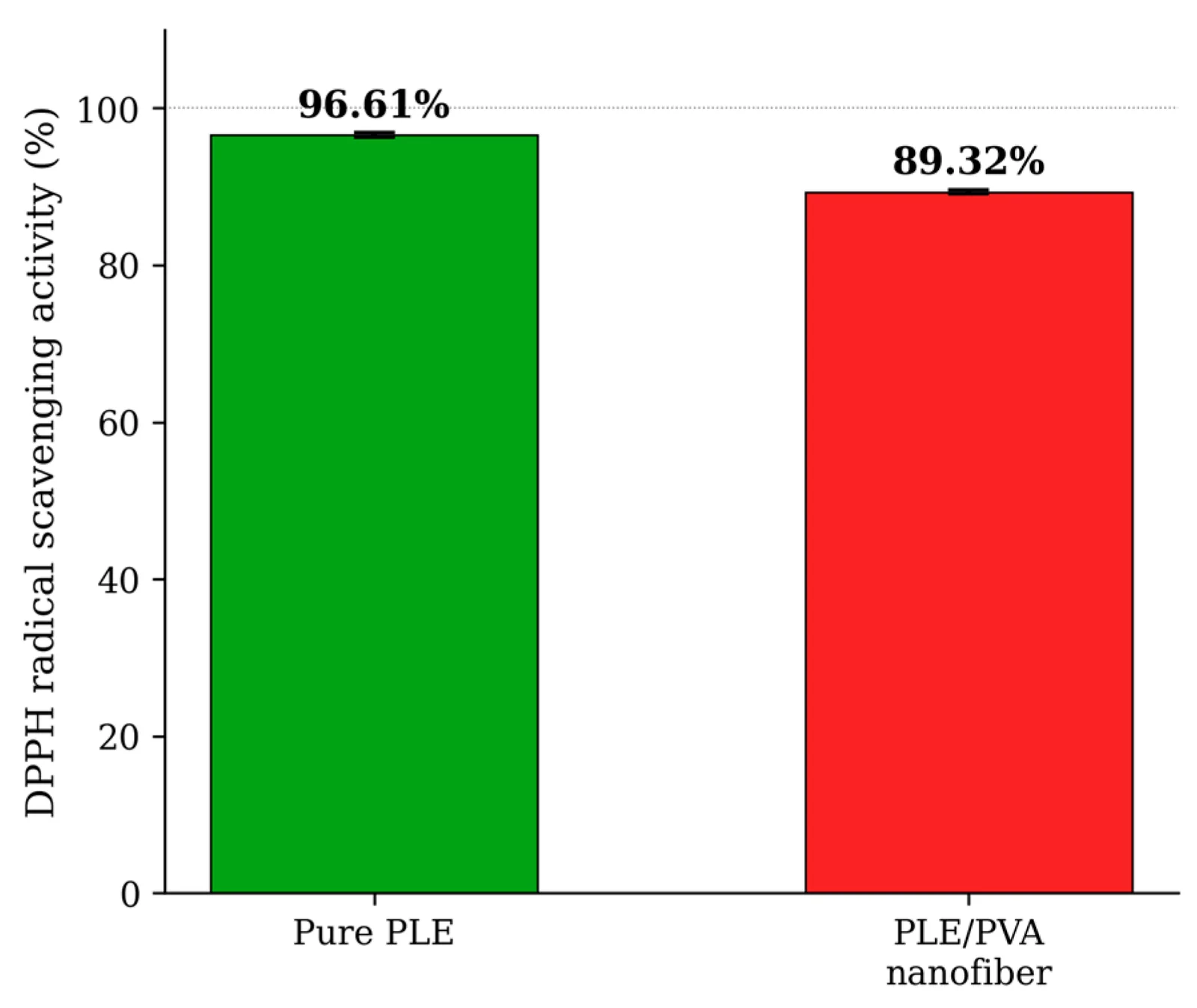
DPPH radical scavenging activity (%) of pure PLE and the electrospun PLE/PVA nanofiber. Data are shown as mean ± SD (*n* = 3).

The antioxidant capacity of the pure PLE and the electrospun PLE/PVA nanofiber was evaluated using the DPPH radical scavenging assay, performed in triplicate (Section 2.5.5). The individual absorbance readings for each sample are reported in Table 5, and the resulting inhibition percentages are shown in Figure 6. The blank DPPH solution yielded a mean absorbance of 0.5117 ± 0.0015. The pure PLE exhibited a high radical scavenging activity of 96.61 ± 0.29% (mean absorbance of 0.0173 ± 0.0015), consistent with its content of phenolic and flavonoid compounds reported for parsley extracts [6,7,25]. Upon incorporation into the PVA matrix, the electrospun PLE/PVA nanofiber showed a radical scavenging activity of 89.32 ± 0.27% (mean absorbance of 0.0547 ± 0.0015), showing that the composite fibers have measurable antioxidant activity. Because the extract loading within the nanofiber was only estimated from the dry-solids content of the extract (Section 2.3) and not measured in the fibers, this value is reported as the experimental inhibition percentage of the PLE/PVA composite and not as the fraction of the original extract activity that was retained. The lower value relative to the free extract may be related (although this was not tested here) to the physical entrapment of the bioactive molecules within the polymer network, which may partially limit their diffusion and direct interaction with the DPPH radical, a trend broadly consistent with other plant-extract-loaded PVA nanofiber systems [33,34]. Because no PVA-only electrospun control was measured here, the contribution of the PVA matrix and of the electrospinning process to the observed activity was not established experimentally; the neat polymer fiber of a comparable electrospun PVA/PVP system has been reported to show negligible radical-scavenging activity [22], and the activity of the composite is therefore attributed to PLE provisionally. Likewise, because the free extract and the nanofiber were not normalized to an equivalent dry extract dose, no reference antioxidant (e.g., ascorbic acid) was included, and possible release of active compounds into the DPPH medium was not examined, the two inhibition values cannot be used to calculate the fraction of activity retained during electrospinning. These results are therefore a first indication of measurable antioxidant activity rather than a full functional characterization; retention efficiency, controlled release, and long-term storage stability will be evaluated in future work. Comparisons of DPPH results are meaningful only under standardized conditions and, ideally, together with other antioxidant assays [35], which were not performed here; in that PVA/PVP system the activity increased with extract concentration [22], which supports the use of dose-normalized comparisons and a polymer-only control in follow-up work. The chemical composition of aqueous parsley leaf extracts, in which flavonoid glycosides such as apiin have been reported as major constituents [36], was not determined for the extract used here, so the activity cannot be assigned to specific compounds.

## 4. Conclusions

In this study, aqueous parsley (*Petroselinum crispum*) leaf extract was successfully incorporated into PVA nanofibers produced by electrospinning. SEM analysis confirmed the formation of continuous submicron fibers under the conditions tested (17 kV, 18 cm, 0.3 mL/h), together with some localized bead-like and film-like features; the mean diameter, based on 40 fiber measurements combined from two separate micrographs, was 333.7 ± 94.0 nm. FTIR results were consistent with hydrogen-bonding interactions between PLE and PVA, although spectral overlap between the two components means this cannot be considered definitive. XRD analysis showed that the main PVA reflection persisted in the composite nanofibers, with a reduction in raw peak intensity in the presence of PLE that is suggestive of a partial reduction in crystallinity. TGA showed that the main decomposition temperature of the PLE/PVA nanofiber (277 °C) fell between that of pure PVA (266 °C) and pure PLE (296 °C), and the composite retained a secondary carbonization step and final residual mass similar to PVA, indicating that PVA dominates the high-temperature degradation behavior of the nanofiber system. The DPPH radical scavenging assay (*n* = 3) indicated that pure PLE and the electrospun PLE/PVA nanofiber exhibited radical scavenging activities of 96.61 ± 0.29% and 89.32 ± 0.27%, respectively, showing that the composite nanofiber has measurable antioxidant activity; because the samples were not normalized to an equivalent extract dose and no PVA-only control was included, the activity retained after electrospinning is not quantified. Together, these results are consistent with the successful incorporation of PLE within a PVA nanofiber matrix without disruption of fiber formation and provide a basic physicochemical characterization of this simple, water-based PLE/PVA system. Parsley extract has recently been combined with PVA and Zn-ascorbate metal–organic frameworks for wound-healing applications [20]; the present study differs in being limited to a simpler, single-component, water-based PLE/PVA system and to its basic physicochemical behavior, and no performance advantage over that or other plant extract-loaded PVA systems is claimed. PVA is a biocompatible, biodegradable polymer and parsley leaf extract is a low-cost, plant-derived source of phenolic compounds; whether this system is suitable for the applications explored for other PVA-based composite materials [14,15,19,37] remains to be tested, as release behavior, antimicrobial activity, mechanical properties, swelling and dissolution behavior, and application-specific performance were not evaluated here. The conclusions are therefore restricted to the physicochemical properties and the preliminary antioxidant activity measured. Future studies should include PVA-only electrospun controls (morphology and DPPH activity), measurement of the extract loading efficiency in the fiber mat, normalization of the antioxidant activity to an equivalent extract dose, antimicrobial activity and release behavior, measurement of solution viscosity and conductivity, a larger fiber diameter distribution from independently prepared batches, confirmation of the crystallinity and thermal analysis results, and evaluation of the long-term stability of the nanofibers under different storage conditions.

## Figures and Tables

**Table 1:** Electrospinning parameter trials examined for PLE/PVA nanofiber optimization in this study. The bold trial number (trial 5) indicates the final conditions used for all characterizations.

Trial No.	PLE:PVA Ratio	PLE (mL)	PVA (mL)	Voltage (kV)	Distance (cm)	Flow Rate (mL/h)
1	80:20	4	1	16–20	12–20	0.1–2
2	60:40	3	2	16–20	12–20	0.1–2
3	50:50	2	2	16–20	12–20	0.1–2
4	40:60	2	3	16–20	12–20	0.1–2
**5**	20:80	1	4	17	18	0.3

**Table 2:** FTIR band assignments for pure PVA, pure PLE, and electrospinning-produced PLE/PVA nanofibers.

Band Assignment	PVA (cm^−1^)	PLE (cm^−1^)	PLE/PVA Nanofiber (cm^−1^)
O–H stretching (hydroxyl groups)	3323.2	3321.8	3314.7
Unassigned (tentative instrumental/atmospheric artifact)	2122.5	2126.7	2123.9
H–O–H bending (adsorbed water)	1636.8	1636.8	1636.8
C–O stretching (ether/ester linkages)	–	1097.3	1100.2
C–C/skeletal vibrations	567.8	604.6	589.0

**Table 3:** XRD peak positions and crystallographic assignments of pure PVA, pure PLE, and electrospinning-produced PLE/PVA nanofibers.

Sample	Main 2θ (°)	d-Spacing (Å)	Additional Reflections, 2θ (°)	Structure
PVA	19.6	4.53	11.3, 14.5, 22.9, 32.1, 36.5, 40.6	Semicrystalline (101 plane)
PLE	–	–	≈9 (broad), ≈20 (broad)	Amorphous
PLE/PVA nanofiber	19.4	4.57	–	Semicrystalline, reduced intensity

**Table 4:** Initial temperature (T(O)), decomposition temperature at maximum weight loss rate (T(Max)) and weight loss (WL) data of pure PVA, pure PLE and PLE/PVA nanofiber produced by electrospinning method according to TGA curves.

Sample	Water Evaporation			Nanofiber Thermal Degradation			Carbonic Residue Decomposition		
	T(O) (°C)	T(Max) (°C)	WL (%)	T(O) (°C)	T(Max) (°C)	WL (%)	T(O) (°C)	T(Max) (°C)	WL (%)
PVA	25	150	0.0	251	266	63.8	400	428	17.1
PLE	25	150	2.2	249	296	49.0	400	715	13.3
PLE/PVA nanofiber	25	150	4.8	259	277	60.7	400	420	11.6

**Table 5:** Individual absorbance readings and calculated DPPH radical scavenging activity for the blank, pure PLE, and PLE/PVA nanofiber (*n* = 3, mean ± SD).

Sample	Reading 1	Reading 2	Reading 3	Mean Absorbance ± SD	DPPH Inhibition (%)
DPPH control (blank)	0.510	0.512	0.513	0.5117 ± 0.0015	–
Pure PLE	0.017	0.016	0.019	0.0173 ± 0.0015	96.61 ± 0.29
PLE/PVA nanofiber	0.053	0.055	0.056	0.0547 ± 0.0015	89.32 ± 0.27

## Data Availability

The data that support the findings of this study are available from the corresponding author upon reasonable request.

## References

[1] Levi S., Rac V., Manojlovic V., Rakic V., Bugarski B., Flock T., Krzycamonik K.E., Nedovi V. (2011). Limonene encapsulation in alginate/poly(vinyl alcohol) fibers obtained by electrospinning. Procedia Food Sci..

[2] Pudziuvelyte L., Petrauskaite E., Stabrauskiene J., Bernatoniene J. (2025). Spray-drying microencapsulation of natural bioactives: Advances in sustainable wall materials. Pharmaceuticals.

[3] Santos J.C.C., Mansur A.A., Ciminelli V.S., Mansur H.S. (2014). Nanocomposites of poly(vinyl alcohol)/functionalized-multiwall carbon nanotubes conjugated with glucose oxidase for potential application as scaffolds in skin wound healing. Int. J. Polym. Mater. Polym. Biomater..

[4] Çeliközlü S. (2025). Use of electrospun chia seed mucilage nanofibers rich in biopolymers in encapsulation of Hypericum perforatum extract. Carbohydr. Polym. Technol. Appl..

[5] Anaya-Mancipe J.M., Queiroz V.M., dos Santos R.F., Castro R.N., Cardoso V.S., Vermelho A.B., Dias M.L., Thiré R.M.S.M. (2023). Electrospun nanofibers loaded with *Plantago major* L. extract for potential use in cutaneous wound healing. Pharmaceutics.

[6] Ahmed W.E., Almutairi A.A., Almujaydil M.S., Algonaiman R., Mousa H.M., Alhomaid R.M. (2025). Nutraceutical potential of parsley (*Petroselinum crispum* Mill.): Comprehensive overview. Ital. J. Food Sci..

[7] Foudah A.I., Alqarni M.H., Alam A., Salkini M.A., Ross S.A., Yusufoglu H.S. (2022). Phytochemical screening, in vitro and in silico studies of volatile compounds from *Petroselinum crispum* (Mill.) leaves grown in Saudi Arabia. Molecules.

[8] Fahami A., Fathi M. (2018). Development of cress seed mucilage/PVA nanofibers as a novel carrier for vitamin A delivery. Food Hydrocoll..

[9] Dehghani S., Noshad M., Rastegarzadeh S., Hojjati M., Fazlara A. (2020). Electrospun chia seed mucilage/PVA encapsulated with green cardamom essential oils: Antioxidant and antibacterial property. Int. J. Biol. Macromol..

[10] Fonseca L.M., dos Santos Cruxen C.E., Bruni G.P., Fiorentini A.M., da Rosa Zavareze E., Lim L.T., Dias A.R.G. (2019). Development of antimicrobial and antioxidant electrospun soluble potato starch nanofibers loaded with carvacrol. Int. J. Biol. Macromol..

[11] Karami N., Kamkar A., Shahbazi Y., Misaghi A. (2021). Electrospinning of double-layer chitosan-flaxseed mucilage nanofibers for sustained release of Ziziphora clinopodioides essential oil and sesame oil. LWT.

[12] Geravand S., Soleimanifard S., Miri M.A., Tavakoli M., Rezaei A. (2025). Fabrication and characterization of olive leaf polyphenols loaded sweet almond gum/gelatin electrospun nanofiber. Food Sci. Nutr..

[13] Amer A.A., Mohammed R.S., Hussein Y., Ali A.S.M., Khalil A.A. (2022). Development of *Lepidium sativum* extracts/PVA electrospun nanofibers as wound healing dressing. ACS Omega.

[14] Zhou Y., Miao X., Lan X., Luo J., Luo T., Zhong Z., Gao X., Mafang Z., Ji J., Wang H. (2020). Angelica essential oil loaded electrospun gelatin nanofibers for active food packaging application. Polymers.

[15] Huang L., Liao R., Bu N., Zhang D., Pang J., Mu R. (2024). Electrospun konjac glucomannan/polyvinyl alcohol long polymeric filaments incorporated with tea polyphenols for food preservations. Foods.

[16] Estrella-Osuna D.E., Tapia-Hernández J.A., Ruíz-Cruz S., Márquez-Ríos E., Ornelas-Paz J.J., Del-Toro-Sánchez C.L., Ocaño-Higuera V.M., Rodríguez-Félix F., Estrada-Alvarado M.I., Cira-Chávez L.A. (2022). Nanoencapsulation of eggplant (*Solanum melongena* L.) peel extract in electrospun gelatin nanofiber: Preparation, characterization, and in vitro release. Nanomaterials.

[17] Stoyanova N., Nachev N., Spasova M. (2023). Innovative bioactive nanofibrous materials combining medicinal and aromatic plant extracts and electrospinning method. Membranes.

[18] Rezaei M., Sedaghat N., Hedayati S., Golmakani M.T. (2024). Fabrication and characterization of novel electrospun nanofibers based on grass pea (*Lathyrus sativus* L.) protein isolate loaded with sumac (*Rhus coriaria* L.) extract. Curr. Res. Food Sci..

[19] Xu X., Zhang Z., Zhu J., Wang D., Liu G., Liang L., Zhang J., Liu X., Li Y., Ren J. (2022). Whey protein isolate nanofibers prepared by subcritical water stabilized high internal phase Pickering emulsion to deliver curcumin. Foods.

[20] Husien S., Haikal R.R., Khalil E.A., El Salakawy N., Abdellatif A., Mamdouh W. (2026). Combined bioactive PVA nanofibrous scaffolds loaded with Zn ascorbate MOFs and *Petroselinum crispum* (*Parsley*) extract for enhanced in-vitro and in-vivo wound healing applications. J. Biol. Eng..

[21] Zeinali T., Alemzadeh E., Zarban A., Khorashadizadeh M., Ansarifar E. (2021). Fabrication and characterization of jujube extract-loaded electrospun polyvinyl alcohol nanofiber for strawberry preservation. Food Sci. Nutr..

[22] Wen P., Hu T.G., Wen Y., Li K.E., Qiu W.P., He Z.L., Wang H., Wu H. (2021). Development of *Nervilia fordii* extract-loaded electrospun PVA/PVP nanocomposite for antioxidant packaging. Foods.

[23] Solaberrieta I., Jiménez A., Cacciotti I., Garrigós M.C. (2020). Encapsulation of bioactive compounds from *Aloe vera* agrowastes in electrospun poly(ethylene oxide) nanofibers. Polymers.

[24] Edikresnha D., Suciati T., Munir M.M., Khairurrijal K. (2019). Polyvinylpyrrolidone/cellulose acetate electrospun composite nanofibres loaded by glycerine and garlic extract with in vitro antibacterial activity and release behaviour test. RSC Adv..

[25] Wong P.Y.Y., Kitts D.D. (2006). Studies on the dual antioxidant and antibacterial properties of parsley (*Petroselinum crispum*) and cilantro (*Coriandrum sativum*) extracts. Food Chem..

[26] Quintero-Borregales L.M., Vergara-Rubio A., Santos A., Famá L., Goyanes S. (2023). Black tea extracts/polyvinyl alcohol active nanofibers electrospun mats with sustained release of polyphenols for food packaging applications. Polymers.

[27] Paynel F., Pavlov A., Colasse L., Rihouey C., Follain N., Duriatti D., Bizet L., Allan I., Lebrun L., Marais S. (2015). Preparation and characterization of flax biocomposites made of seed mucilage reinforced by fibers. Compos. Part A Appl. Sci. Manuf..

[28] Dağcı Tekin M. (2026). Production, Characterization and Antioxidant Activity of Electrospun Nanofibers Using Nettle (*Urtica dioica* L.) Seed Mucilage-Polyvinyl Alcohol. Polymers.

[29] Yang S.B., Kim E.H., Kim S.H., Kim Y.H., Oh W., Lee J.T., Jang Y.A., Sabina Y., Ji B.C., Yeum J.H. (2018). Electrospinning fabrication of poly(vinyl alcohol)/Coptis chinensis extract nanofibers for antimicrobial exploits. Nanomaterials.

[30] Zhang W., Ronca S., Mele E. (2017). Electrospun nanofibres containing antimicrobial plant extracts. Nanomaterials.

[31] Rasheed M.H., Mousa M.H., Kadhim Q.S., Abdelmoula N., Khalfallah A., Benzarti Z. (2025). Novel electrospun PVA-PVP-PAAm/TiO2 nanofibers with enhanced optoelectrical, antioxidant and antibacterial performances. Polymers.

[32] METTLER TOLEDO Interpreting TGA Curves. Thermal Analysis Application No. UC 131, UserCom 13. https://www.mt.com/gb/en/home/supportive_content/matchar_apps/MatChar_UC131.html.

[33] Sadri M., Hosseini H., Pezeshki-Modaress M., Zandi M. (2015). Structural and morphological properties of electrospun green tea/poly(vinyl alcohol) nanofibers. J. Appl. Polym. Sci..

[34] Aydoğdu A., Sumnu G., Sahin S. (2019). Fabrication of peppermint and rosemary essential oil loaded electrospun PVA/SA nanofibers. Food Packag. Shelf Life.

[35] Gulcin İ., Alwasel S.H. (2023). DPPH radical scavenging assay. Processes.

[36] Epifanio N.M.M., Cavalcanti L.R.I., dos Santos K.F., Duarte P.S.C., Kachlicki P., Ożarowski M., Riger C.J., Chaves D.S.A. (2020). Chemical characterization and in vivo antioxidant activity of parsley (*Petroselinum crispum*) aqueous extract. Food Funct..

[37] Rajamani S., Gani R.S., Kasai D.R., Ashok A., Anjanayya G., Kamble A.A. (2026). Fluconazole NP-Loaded PVA/Chitosan films for food packaging. Chem. Pap..

